# Treatment of localized prostate cancer and use of nomograms among urologists in the West Africa sub-region

**DOI:** 10.11604/pamj.2020.36.251.21419

**Published:** 2020-08-06

**Authors:** Mathew Yamoah Kyei, Ben Adusei, George Oko Klufio, James Edward Mensah, Samuel Gepi-Attee, Emmanuel Asante

**Affiliations:** 1 Department of Surgery and Urology, University of Ghana Medical School, Accra, Ghana,; 2 Department of Surgery, 37 Military Teaching Hospital, Accra, Ghana

**Keywords:** Localized prostate cancer, nomogram, treatment, West Africa

## Abstract

**Introduction:**

there is a high incidence of prostate cancer among men of African descent. The disease tends to occur at an early age with a tendency to be aggressive. The objective was to determine the practice of urologists in the West African sub-region regarding treatment of localized prostate cancer, the use of nomograms and their perception of the usefulness of nomograms.

**Methods:**

this was a cross-sectional study that involved urologists practicing in the West African sub-region attending urology and surgery conferences of the “Société Internationale d´Urologie”, West African college of surgeons and the Ghana association of urological surgeons. A structured questionnaire was used that sort to ascertain the treatment modalities used for localized prostate cancer and the use of nomograms in the sub-region. The study period spanned the years 2018 and 2019.

**Results:**

fifty-six urologists practicing in eleven West African countries responded. Fifty percent had been in practice for less than 5 years. Sixty eight percent (38/56) had been involved in the treatment of localized prostate cancer. Radical prostatectomy was widely available and the treatment modality most used 94.7% (36/38). Nomograms was used by 57.9% of them (22/38) with the Partin tables being the most commonly used nomogram (34.2%). No Locally developed nomogram for treatment of localized prostate cancer was identified.

**Conclusion:**

radical prostatectomy is the commonest treatment modality used for the management of localized prostate cancer in the West Africa sub-region. Majority of the urologists used nomograms with the Partin tables being the most used.

## Introduction

Prostate cancer is one of the most commonly diagnosed cancers in men and a major cause of cancer death among them. There is a high incidence among men of African descent-234.6/100,000 [1,2]. The Incidence on the African continent vary substantially by sub-region, with rates highest in the east, intermediate in the south, and lowest in the west [3]. In a study in Ghana, the screen detected rate was about 7% [4,5] and in Nigeria it was 1046/100,000 [6]. It has been noted that in people of African ancestry, the disease tended to occur at an early age and also tended to be aggressive [2]. Thus, early detection and timely intervention will be of benefit in these populations. It has been observed that patients with prostate cancer in the West Africa sub-region present with advanced disease with less than 43% (6.4-43%) reporting with localized prostate cancer [7], in contrast to higher income countries where 80-90% present with localized prostate cancer [7,8]. There has been an increase in screening for prostate cancer using PSA and digital rectal examination in West Africa in recent times, especially in the urban centers [4]. This is anticipated to lead to an increase in the detection of localized prostate cancer requiring the need for curative treatment for selected patients with low risk disease and those with an intermediate or high risk disease [7]. This is achieved using radical prostatectomy, external beam radiotherapy and brachytherapy with the need for anti-androgens in those with intermediate and high-risk disease [9]. Cryotherapy and high intensity focused ultrasound are other modes of treatment that could be considered.

Various publications have indicated the increasing use of curative procedures for localized prostate cancer in the West African sub-region such as radical prostatectomy, external beam radiation therapy and brachytherapy [4,7,10-12]. The decision-making process leading to the choice of a suitable method for curative treatment could be challenging. This is because it requires a fine balance between expected clinical benefit, life expectancy, comorbidities and potential treatment-related adverse events [13]. Various guidelines such as those by the American Urological Association (AUA), the European Urological Association (EAU) and the National Comprehensive Cancer Network (NCCN) give stratifications that provide guidance for decision making in the management of localized prostate cancer. This includes active surveillance for very low risk and low risk localized prostate cancer which is reported to have a less satisfactory outcomes in blacks due to the aggressive nature of the disease in this population sub group compared to Caucasians [7,14]. The review by Cassell A *et al*. indicate that the use of this stratification was far from the reality from publications on management of localized prostate cancer in the African region [7]. In addition to risk groupings [15], various other nomograms/predictive tools have been developed to help in the prognostication of the outcomes of treatment that also sake to guide the choice of treatment for localized prostate cancer and for patient counselling. These predictive tools include Kattan-type nomograms [16], artificial neural networks (ANNs) [17], probability tables [18], classification and regression tree (CART) analyses [19], probability formulas, look-up and propensity scoring tables [13].

These use individualized variables to generate probability of organ confinement which is based on models so as to personalize treatment and prognostication. There is increase development and rise in the use of nomograms in the clinical decision-making process for treatment of cancers [20]. They have been noted to be better than conventional use of the TNM staging in its application as they take into consideration both biological and clinical variables and assign points that are based on the contribution of the variable to overall prognosis. Other advantages of nomograms include the inclusion of continuous variables and relevant determinants of disease into prognosis [21]. It is also user friendly and superior to clinical judgement in predicting disease course [22]. The availability of internet-based versions allows patients to input the relevant data and become informed on various aspects of their disease process allowing them to contribute to the decision-making process. These versions also facilitate their use by clinicians as inputted data are readily displayed in real time. This study sort to find the experiences of urologists practicing in the West Africa sub-region on their management of patients with localized prostate cancer, the availability of various treatment options in their country of practice based on referral patterns and their use of nomograms/prediction tools in the management of these cases. Their perception of the usefulness of these nomograms/prediction tools was also determined. It was also to ascertain if there was a locally developed nomogram that take into consideration the peculiarities of the African sub-population.

## Methods

This was a cross-sectional questionnaire-based study involving urologists practicing in the West Africa sub-region attending urology and surgery conferences of the societe internationale d´ urologie, West African college of surgeons and Ghana association of urological surgeons. A structured questionnaire was used. The study period spanned the years 2018 and 2019. The responses of interest included how long they had been practicing as urologists, the country of practice, the available treatment modalities for patients with localized prostate cancer in their country of practice based on their referral patterns. Also determined was their personal involvement in the treatment of localized prostate cancer, the modalities used for treatment and the type of nomograms used. Their perception of the usefulness of nomograms in the treatment of localized prostate cancer was also determined. The present study protocol was reviewed and approved by the institutional review board of the institutional ethical committee of the 37 Military Hospital in Accra, Ghana (approval no: 37MH-IRB-IPN 263/2018). Informed consent was obtained from all subjects when they were enrolled. The results were analyzed using the SPSS version 21 and presented as descriptive statistics and tables.

## Results

A total of 56 specialist/consultant urologists working in eleven West African countries responded Table 1. For the number of years of practice, 28/56 (50.0%) had been in practice for less than 5 years, 11/56 (19.6%) had practiced for 5-9 years and 17/56 (30.4) for 10 years or longer. Majority of the respondents 87.5% (49/56) had referred or managed a patient with localized prostate cancer in the last one year. Radical prostatectomy was the widely available treatment modality in the sub-region that patients were likely to receive for localized prostate cancer. It was available in nine of the eleven countries represented in the study. Brachy therapy was available in only three of the countries represented Table 2. With regards to their being personally involved in the treatment of localized prostate cancer in the past year, 67.9% (38/56) had been personally involved while the remainder had not. Radical prostatectomy was the treatment modality most used by these urologists 94.7% (36/38) which corresponded to 64.3% (36/56) of the respondents. The use of external beam radiotherapy and brachytherapy were 34.2% (13/38) and 15.8% (6/38) respectively. Of those who had been personally involved in the treatment of localized prostate cancer within the past one (1) year, 57.9% (22/38) used a nomogram. Partin tables was the most frequently used nomogram (34.2%; 13/38) with two (5.3%) using the memorial Sloan Kettering cancer center nomogram. Others such as the D´Amico, and TNM classification were indicated. Nomograms were not used by 42.1% (16/38). In response to whether nomograms were useful for management of localized prostate cancer, 21.4% (12/56) strongly agreed, 44.6% (25/56) agreed, 19.6% (11/56) somehow agreed, while 3.6% (2/56) disagreed. Six (10.7%) respondents offered no response. No locally developed nomogram for the management of localized prostate cancer was identified.

**Table 1:** country of practice of the urologists

Country	No. of urologists (%)
Ghana	22(39.3)
Nigeria	14(25)
Burkina Faso	6(10.7)
Senegal	5(8.9)
Benin	2(3.6)
Niger	2(3.6)
Cameron	1(1.8)
Chad	1(1.8)
Mali	1(1.8)
Mauritania	1(1.8)
Sierra Leone	1(1.8)
Total	56(100.0)

**Table 2:** country distribution of available primary treatment modalities for patients with localized prostate cancer in the West Africa sub-region

	Available treatment modality		
Country	Radical Prostatectomy	External beam Radiotherapy	Brachytherapy
Ghana	Yes	Yes	Yes
Nigeria	Yes	Yes	Yes
Burkina Faso	Yes	Yes	Yes
Senegal	Yes	Yes	No
Benin	Yes	Yes	No
Niger	Yes	No	No
Cameroon	Yes	Yes	
Chad	Yes	No	No
Mali	-	-	-
Mauritania	Yes	Yes	No
Sierra Leone	No	No	No

## Discussion

It has been noted that between 6.4-43% of patients in the African sub-region present with localized prostate cancer [7]. However, with the increasing use of PSA screening, these numbers are expected to increase. As noted, majority of the urologists (87.5%) had referred or managed a patient with localized prostate cancer in the last one year. This support other publications that indicate that despite the majority of patients with prostate cancer presenting with advanced disease in the sub-region, there are significant numbers that present with localized disease [10-12] making it an important disease condition requiring to be researched into and whose management need to be improved in the West Africa sub-region. The availability of primary treatment modalities namely, radical prostatectomy, external beam radiotherapy and brachy therapy varied widely among the countries in which the urologists practiced based on their referral patterns. All three modalities were available in only three of the countries. Of the remaining countries, four and two were equipped with two and one of the treatment modalities respectively. One of the eleven countries represented had no treatment modality. This study found that radical prostatectomy was the most widely available treatment modality for localized prostate cancer. It was also the commonest treatment option employed by the urologists with 64.3% of the respondents having been part of a procedure of radical prostatectomy in the past year. There is the need to train more urologists in the sub-region so they acquire the technical skill to perform radical prostatectomy as it has been noted that patients with localized prostate cancer with indication for radical prostatectomy may not get the needed treatment in Africa due to the technical nature of the procedure, lack of access and finance [7].

The findings that 50% of the respondents had practiced for less than 5 years is indicative of a relatively less experienced population of urologists practicing in the West African sub-region who can be considered as fertile for transfer of the skill for radical prostatectomy. Some countries in the sub-region had few or no urologists practicing in the country as reflected in the country distribution of the specialists. The use of brachy therapy among the urologists was low in the sub-region with only three countries; Ghana, Burkina Faso and Nigeria reporting its availability. No focal therapy such as cryotherapy or high intensity ultrasound therapy was reported in this current study and is similar to findings by Rebbeck *et al*. in 2011 (8 years earlier) showing a rather low uptake of these modalities in the West Africa sub-region [23]. On the use of nomograms, 57.9% of the urologists used nomograms in counselling and decision making in the treatment with the Partin tables commonly used in the sub-region (34.2%). The Partin tables [18] developed mainly with people of European descent might not perform as well in people of African descent. In a study by Yadav *et al*. using a cohort of Indian population, they found that though the Partin tables (2007) had a fair discriminatory property, it did not accurately predict lymph node involvement, seminal vesicle invasion and extracapsular extension across the entire range of predicted values [24]. There is therefore a need for a study to determine the Partin tables predictive performance among the West African population as it is the most commonly used nomogram in the treatment of localized prostate cancer in the sub-region. Most of the urologists (85.7%) agreed that nomograms and prediction tools were useful in decision making and counselling. No locally developed nomogram for the management of localized prostate cancer in the West African sub-region, taking into consideration the racial influence and tumour biology and characteristics, was identified.

## Conclusion

Majority of urologists practicing in the West Africa sub-region encounter cases of localized prostate cancer requiring curative intervention. Radical prostatectomy is the commonest treatment modality available for the treatment of prostate cancer in the sub-region. Majority of the urologists agreed that the use of nomograms improved the decision making in managing these cases with the Partin tables being the most used. No locally developed nomogram for treatment of localized prostate cancer was identified.

### What is known about this topic

- Prostate cancer is common in people of African descent and it tends to be aggressive;
- Majority present with advanced disease;
- Availability of curative treatment for localized prostate cancer in the African continent such as radical prostatectomy, external beam radiation and brachytherapy.

### What this study adds

- This study reports that 87.5% of urologists practicing in the West African sub-region have managed localized prostate cancer within the preceding year;
- It provides current country distribution of various treatment options for localized prostate cancer in eleven West African countries;
- Nomograms are used by urologists in the management of localized prostate cancer with the Partin tables being the most used. No locally developed nomogram was identified.
